# Bone Phenotype in Autosomal Dominant Polycystic Kidney Disease

**DOI:** 10.1007/s00223-025-01471-w

**Published:** 2026-01-23

**Authors:** Magdalena Jankowska, Per Magnusson, Malgorzata Debowska, Bengt Lindholm, Abdul Rashid Qureshi, Tomasz Lukaszuk, Alicja Dębska-Ślizień, Daniel Guido Fuster, Peter Stenvinkel, Mathias Haarhaus

**Affiliations:** 1 Department of Nephrology, Transplantology and Internal Medicine, Medical University of Gdańsk, Gdańsk, Poland; 2 Department of Clinical Chemistry, Department of Biomedical and Clinical Sciences, Linköping University, Linköping, Sweden; 3 Nalecz Institute of Biocybernetics and Biomedical Engineering, Polish Academy of Sciences, Warsaw, Poland; 4 Renal Medicine, Department of Clinical Science, Intervention and Technology, Karolinska Institutet, Stockholm, Sweden; 5 Faculty of Computer Science, Bialystok University of Technology, Bialystok, Poland; 6 Department of Nephrology and Hypertension, Inselspital, Department for BioMedical Research (DBMR), Bern University Hospital, University of Bern, Bern, Switzerland

**Keywords:** Autosomal dominant polycystic kidney disease, Bone-specific alkaline phosphatase, Tartrate-resistant acid phosphatase isoform 5b, Intact procollagen type i n-terminal propeptide, Chronic kidney disease, Renal osteodystrophy

## Abstract

Autosomal dominant polycystic kidney disease (ADPKD) is a systemic ciliary disorder, yet the impact on bone health remains insufficiently defined. Given the expression of polycystins in osteocytes, osteoclasts and extracellular matrix, a disease-specific bone phenotype has been hypothesized. This study aimed to comprehensively characterize the bone phenotype in ADPKD across stages of chronic kidney disease (CKD) and Mayo Imaging Classification (MIC). In this cross-sectional study, 81 patients with ADPKD, 15 with CKD of other etiologies, and 21 healthy individuals were included. Bone phenotype was assessed across four domains: bone metabolism biomarkers, bone turnover biomarkers, bone mass/microarchitecture (dual-energy X-ray absorptiometry with trabecular scoring (TBS)) and bone material strength (microindentation, BMSi). Z-score-based heat-maps were used to visualize stage-specific alterations, and multivariable regression analysis was applied to estimate model parameters. ADPKD patients consistently showed lower bone turnover, particularly PINP and in CKD G1-2 also BALP, while BMSi, BMD and TBS did not differ from healthy individuals. Across CKD stages, mineral abnormalities reflected the effect of declining eGFR, whereas stratification by MIC revealed less distinct patterns, indicating that the bone phenotype may be more closely linked to CKD progression than cystic burden in later CKD stages. This first comprehensive analysis of the ADPKD bone phenotype reveals mineral disturbances, reduced bone turnover, and preserved bone mass and microarchitecture in early ADPKD. Bone alterations followed kidney function rather than MIC stage. Impact microindentation, BMSi, did not reveal any distinctive pattern in ADPKD.

**Supplementary Information:**

The online version contains supplementary material available at 10.1007/s00223-025-01471-w.

## Introduction

Autosomal dominant polycystic kidney disease (ADPKD) is a systemic disorder with a wide range of extrarenal manifestations, including effects on bone health [1–3]. The clinical course of the disease shows significant variation between individuals, with disease progression primarily determined by the dosage of polycystin, a protein whose structural integrity and functional capacity are governed by multiple genetic factors [4]. The progression of the disease in the kidneys can be quantitatively assessed through total kidney volume (TKV), which advances in a stepwise manner constrained by the so-called “cystogenesis threshold” [5, 6]. Conversely, in extrarenal manifestations, including those affecting osseous tissue, the phenotypic expression appears to occur along a continuum rather than as distinct pathological transitions, a hypothesis explored in this study.

Assessing bone health in ADPKD presents a complex challenge, as it is affected by an interplay of factors, including tissue mineralization, organic matrix composition, micro- and macro-architecture, intrinsic material properties, and remodeling activity. The most definitive manifestation of impaired biomechanical competence is fracture, a concerning event observed with increased prevalence in individuals affected by ADPKD [1].

The skeletal phenotype in ADPKD is increasingly recognized as distinct from that observed in other chronic kidney diseases [2]. Accumulating evidence points to a characteristic profile marked by low bone turnover, preserved cortical bone mineral density (BMD), and altered biochemical markers. Notably, patients consistently exhibit elevated circulating levels of bioactive sclerostin and fibroblast growth factor 23 (FGF23), alongside reduced total alkaline phosphatase (ALP) [3, 4]. This constellation of findings suggests a unique bone remodeling pattern, potentially reflecting disease-specific mechanisms that diverge from classical CKD-associated mineral and bone disorders (CKD-MBD).

**Fig. 1:**
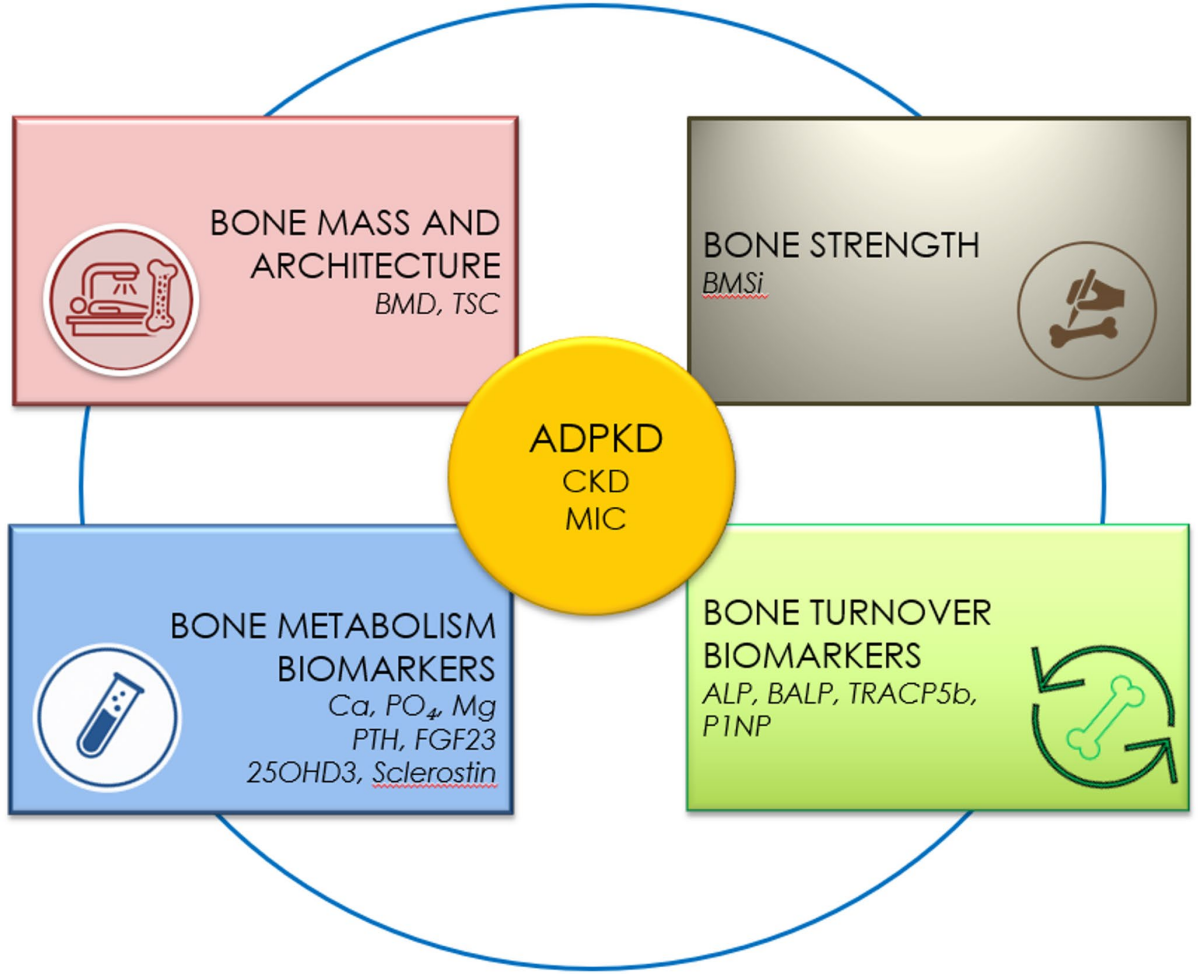
Model of proposed integrative approach to bone phenotype in autosomal dominant polycystic kidney disease (ADPKD) as a model of chronic kidney disease (CGA classification). The framework integrates four complementary domains: bone mass and architecture (bone mineral density, BMD; trabecular score, TSC), bone material strength (bone material strength index, BMSi), bone metabolism biomarkers (calcium, Ca; phosphate, Pi; magnesium, Mg; parathyroid hormone, PTH; fibroblast growth factor 23, FGF-23; 25-hydroxyvitamin D₃, 25OHD3; bioactive sclerostin), and bone turnover biomarkers (alkaline phosphatase, ALP; bone-specific alkaline phosphatase, BALP; tartrate-resistant acid phosphatase isoform 5b, TRACP5b; intact procollagen type I N-terminal propeptide, PINP). Disease progression assessment integrates the CGA (cause–glomerular filtration rate–albuminuria) classification of CKD and the Mayo Imaging Classification (MIC) for ADPKD

While bone biopsy remains the gold standard for direct diagnostic evaluation, especially in the context of CKD-MBD [5], it is not without limitations, including invasiveness, costs, and restricted accessibility and several reference bone turnover markers have recently been suggested as alternative indicators of bone turnover that can be used in patients with CKD [6]. This study introduces a novel strategy designed to improve the assessment of bone health in ADPKD, offering a comprehensive and refined approach to understanding skeletal involvement in this disease (Fig. 1).

The study aims were: (1) to describe the bone phenotype of ADPKD in comparison to other etiologies of CKD and healthy individuals; (2) to evaluate whether the bone phenotype of ADPKD differs between consecutive stages of CKD; (3) to evaluate whether the bone phenotype of ADPKD differs between consecutive stages of ADPKD.

## Materials and Methods

### Study Population

This study included 117 participants (M 62/ F 55), recruited from the University Clinical Hospital of Gdańsk, a tertiary reference center in Poland, between March 2021 and June 2022. The cohort comprised 81 patients diagnosed with ADPKD and 15 patients with CKD of other etiologies, including IgA nephropathy or other/unknown primary diseases, excluding diabetes mellitus (DM). The control group consisted of 21 individuals, recruited from the general population of the region, who self-reported as healthy. All participants were adults and underwent screening for eligibility based on predefined inclusion and exclusion criteria specific to their respective groups. Diagnosis of ADPKD was confirmed according to clinical criteria, Ravine-Pei [7].

ADPKD and CKD group’s exclusion criteria: lack of informed consent; diagnosis of DM as an underlying cause of CKD or as a comorbidity, due to anticipated alterations in mineral and bone metabolism; current or previous immunosuppressive therapy, including corticosteroids, owing to their known impact on bone phenotype; estimated glomerular filtration rate (eGFR) < 15 ml/min/1.73 m², indicative of advanced CKD-associated mineral and bone disorder; known allergy to local anesthetic agents required for bone material strength index (BMSi) assessment; presence of leg oedema or obesity, which may interfere with probe penetration and compromise the accuracy of BMSi measurements.

Healthy volunteers’ exclusion criteria: lack of informed consent, known allergy to local anesthetic agents required for BMSi assessment; presence of leg oedema or obesity, which may affect probe penetration and the accuracy of BMSi measurements.

Further stratification of ADPKD participants was based on the stages of CKD according to the Kidney Disease: Improving Global Outcomes (KDIGO) guidelines [8] and the stages of Mayo Clinic Imaging Classification (MIC) [9]. The Chronic Kidney Disease Epidemiology Collaboration (CKD-EPI) equation was used to estimate GFR from measured serum creatinine levels [10].

Class 1 ADPKD patients were stratified into five subclasses based on age and height-adjusted total kidney volume (HtTKV) assessed with magnetic resonance imaging (MRI). According to these parameters, Class 1 subgroups named A, B, C, D and E are identified, and the risk for GFR deterioration is assumed to increase progressively from Class 1 A to Class 1E [9].

### Material

Fasting venous blood and urine samples were collected. The biological material was centrifuged immediately and stored (plasma and serum) at − 70 °C until analysis.

Serum samples of creatinine, albumin, calcium, phosphate, magnesium, intact parathyroid hormone (PTH), vitamin D, high-sensitivity C-reactive protein (CRP) and ALP were measured by routine automated methods at the certified Central Clinical Laboratory, University Clinical Center, Gdańsk, Poland.

Bone biomarkers were analyzed at the Department of Clinical Chemistry (Swedac accredited no. 1342), Linköping University Hospital, Linköping, Sweden. All samples were assayed with reagents from the same batch. Serum bioactive sclerostin was measured by enzyme-linked immunosorbent assay (ELISA) (Biomedica, Vienna, Austria), intact fibroblast growth factor-23 (FGF23) by ELISA (Kainos Laboratories, Inc., Tokyo, Japan) and C-terminal FGF23 by ELISA (Biomedica, Vienna, Austria). Both serum BALP and TRACP5b were analyzed with chemiluminescent immunoassays assays on the iSYS fully automated instrument (Immunodiagnostic Systems, Ltd., Boldon, UK). Serum intact PINP was measured with the UniQ radioimmunoassay (Aidian Oy, Espoo, Finland).

### MRI Protocol and HtTKV Calculation

MR Siemens Magnetom Aera 1,5T (Siemens Healthineers Global) was used to perform MRI. The protocol based on standard guidelines does not require contrast medium injection [10]. Data were analyzed using Syngo.via MR Siemens software.

### Densitometric Measurements

Areal bone mineral density (aBMD) was measured by dual-energy X-ray absorptiometry (DXA) in four regions: lumbar spine, proximal femur, non-dominant forearm and whole body. Hologic Discovery Wi bone densitometer was used for this purpose (Hologic, Inc., Marlborough, MA, USA). A standard protocol for positioning was used to reduce aBMD variability. Results were reported according to the International Society for Clinical Densitometry recommendations by one clinician [11]. Lumbar spine scans were re-analyzed using TBS iNsight computer software (Medimaps, Merignac, France version 3.0.2) to calculate trabecular bone score (TBS).

### Bone Mechanical Properties

The bone mechanical properties were assessed using OsteoProbe^®^ (Active Life Scientific, Santa Barbara, CA, USA) to measure the BMSi. Following local anesthesia, the handheld OsteoProbe^®^ was inserted through the skin at the midshaft of the right tibia, positioned at the mean distance between the distal apex of the patella and the medial malleolus. The device was advanced until it reached the bone surface, which was indented upon activation.

A minimum of 8 to 10 measurements were performed from a single skin insertion site in a clockwise direction. After completing bone measurements, additional measurements were conducted on a polymethylmethacrylate (PMMA) plastic calibration phantom. The BMSi outcome measure was calculated as 100 times the ratio of the indentation distance in the PMMA standard divided by the indentation distance in bone.

### Statistical Analysis

Quantitative and categorical variables were presented as medians with interquartile ranges and numbers with percentages, respectively. Patients were categorized into various groups and compared. The Wilcoxon rank sum test and the chi-squared test were used to identify statistically significant differences between two groups for quantitative and categorical variables, respectively. The Kruskal-Wallis test and the chi-squared test were used to identify statistically significant differences between three groups for quantitative and categorical variables, respectively.

Two complementary heat maps were generated to visualise the distribution of mineral and bone parameters across disease stages within the ADPKD cohort only. The first heat map was based on CKD staging reflecting disease progression according to kidney function (eGFR). The second was constructed according to the MIC, which stratifies patients morphologically based on HtTKV. This approach allowed parallel visualisation of the skeletal phenotype along both functional and structural dimensions of ADPKD progression. It enabled us to identify domain-specific patterns (bone mass, bone strength, biomarkers, bone turnover) and compare their distribution between two classifications. Z-scores were computed separately for each variable, based on its own mean and standard deviation within the analysed cohort. Z-scores are not absolute differences, and are intended to give an overview of patterns rather than to indicate statistical significance. Accordingly, the color intensity in the heat maps represents the relative deviation of a given parameter from its mean, not absolute values. As a result, similar colors may correspond to different absolute Z-scores across variables, reflecting independent scaling of each parameter.

We applied a rank-based linear regression method. Compared to conventional linear regression, rank-based regression is inherently more robust, as outlying observations exert a far weaker influence on the resulting model. Estimation of the regression coefficients was carried out by minimizing the convex and piecewise linear (CPL) regression-rank criterion function, as proposed by Bobrowski [12, 13]. Normalized feature importance scores (0–100%, summing to 100%) were calculated to reflect each variable’s influence. Conceptually, this approach maximizes the probability that, when comparing two patients within the ADPKD cohort, the model will correctly identify which one presents with a more advanced disease stage. The analysis was carried out across three distinct groups designated as ranks 1, 2, and 3, respectively: CKD G1-2, CKD 3a, and CKD G3b-4, according to the CGA; and Mayo A-B, C, and DE, according to the MIC classification.

Statistical analyses were performed using R version 4.5.1 and own implementation in Python 3.9 environment for the ranked regression CPL method. Results were considered statistically significant at a *p *value < 0.05, unless otherwise indicated.

## Results

Table 2 presents the demographic and clinical characteristics of participants. Groups were well-balanced according to demography. As expected, kidney function markers differed: serum creatinine and albumin-to-creatinine ratio (ACR) were higher in the CKD group compared with both ADPKD and healthy controls. No other significant differences were observed. ADPKD patients were predominantly in CKD stage 3 or higher, with distribution across Mayo imaging classification (MIC) stages 1B–1E, while the group of CKD patients with other etiologies than ADPKD was almost solely restricted to stage 3.

**Table 1:** Characteristics of the study group

	ADPKD (*n* = 81)	Non-ADPKD CKD (*n* = 15)	Healthy (*n* = 21)
Sex, F/M	35/46 (43.2%/56.8%)	7/8 (46.7%/53.3%)	13/8 (61.9%/38.1%)
Age, year	44.00 [34.00, 50.00]	41.00 [37.50, 43.50]	42.00 [33.00, 46.00]
BMI, kg/m^2^	25.10 [23.15, 28.65]	23.81 [22.27, 28.30]	24.73 [23.48, 28.14]
Creatinine, mg/dL	1.33 [0.93, 1.80]	1.73 [1.61, 1.99]	0.80 [0.71, 0.91]
eGFR, mL/min/1.73 m^2^	58.00 [41.00, 90.00]	39.00 [32.50, 44.50]	> 60.00
ACR, mg/g	20.31 [9.53, 71.22]	192.79 [24.74, 736.91]	3.99 [2.37, 4.94]
CRP, mg/dL	1.14 [0.59, 2.66]	1.02 [0.80, 1.56]	0.74 [0.30, 1.45]
Vitamin D supplementation, yes/no	30/51 (37.0%/63.0%)	7/8 (46.7%/53.3%)	4/17 (19.0%/81.0%)
HCO3^−^, mmol/L	23.40 [22.50, 24.40]	22.00 [20.75, 22.95]	23.90 [23.10, 24.70]
CKD stages			
G1	21 (25.9%)	0 (0.0%)	19 (90.5%)
G2	16 (19.8%)	1 (6.7%)	2 (9.5%)
G3	34 (42.0%)	11 (73.3%)	0 (0.0%)
G4	10 (12.3%)	3 (20.0%)	0 (0.0%)
MIC stages			
1 A	3 (3.70%)	–	–
1B	10 (12.35%)	–	–
1 C	17 (20.99%)	–	–
1D	33 (40.74%)	–	–
1E	18 (22.22%)	–	–

### Mineral and Bone Phenotype in Early ADPKD Compared To Healthy Individuals

Individuals with ADPKD and preserved kidney function showed lower levels of the bone formation markers BALP and PINP, lower serum phosphate and magnesium, and higher urinary ACR compared to healthy individuals, whereas there were no significant differences in terms of age, sex, BMI, serum creatinine, or CRP levels (Table 2). Morphological and functional indicators of bone health, namely aBMD, TBS, and BMSi, were not significantly affected in ADPKD with preserved kidney function compared to healthy individuals (Table 2). The violin plots presenting variables showing significant differences are available in supplementary material (Fig. S1).

### Mineral and Bone Phenotype in ADPKD Compared To Other CKD Etiologies

Compared with patients with CKD stages G3-4 of other etiologies, ADPKD patients with CKD G3-4 had lower levels of urinary ACR, higher levels of bioactive sclerostin and lower levels of PINP. Median BALP and TRAcP5b were also lower but did not achieve statistical significance. Detailed results are provided in Table 3. The violin plots presenting variables showing significant differences are available in supplementary material (Fig. S2).

**Table 2:** Clinical data and mineral and bone phenotype in ADPKD with preserved kidney function (CKD G1-G2) vs. healthy

		ADPKD CKD G1-G2 (*n* = 37)	Healthy (*n* = 21)	*P* value
	Age, year	36 [31,47]	42 [33,46]	0.583
Clinical data	Sex, F/M	21/16 (56.8%/43.2%)	13/8 (61.9%/38.1%)	0.916
	BMI, kg/m^2^	24.17 [22.31, 25.93]	24.73 [23.48, 28.14]	0.148
	Creatinine, mg/dL	0.91 [0.78, 1.06]	0.80 [0.71, 0.91]	0.070
	ACR, mg/g	15.19 [7.78, 32.89]	3.99 [2.37, 4.94]	**< 0.001**
	CRP, mg/dL	1.02 [0.49, 2.47]	0.74 [0.30, 1.45]	0.201
Bone metabolism	Calcium, mg/dL	9.50 [9.30, 9.90]	9.70 [9.50, 9.80]	0.548
	Phosphate, mg/dL	3.25 [2.80, 3.40]	3.40 [3.20, 3.70]	**0.026**
	Magnesium, mg/dL	1.90 [1.80, 2.00]	2.00 [2.00, 2.10]	**0.008**
	PTH, pg/mL	34.40 [21.00, 45.10]	30.00 [23.70, 32.60]	0.387
	FGF23 intact, pg/mL	52.05 [34.92, 64.95]	45.86 [34.26, 64.65]	0.663
	FGF23 C-terminal, RU/mL	1.04 [0.43, 2.04]	0.85 [0.61, 1.20]	0.374
	Bioactive sclerostin, pmol/L	45.27 [30.74, 62.50]	48.26 [36.76, 75.99]	0.601
	25OHD3, ng/mL	25.87 [20.12, 32.49]	27.11 [21.64, 35.25]	0.561
Bone mass/ microarchitecture	aBMD wb, g/cm^2^	1.13 [1.08, 1.18]	1.13 [1.07, 1.21]	0.939
	TBS	1.45 [1.38, 1.51]	1.45 [1.39, 1.50]	0.950
Bone strength	BMSi	76.10 [64.60, 81.00]	77.90 [71.70, 81.90]	0.544
Bone turnover	ALP, U/L	53.00 [43.00, 63.00]	56.00 [48.00, 71.00]	0.182
	BALP, µg/L	10.87 [9.75, 14.99]	14.88 [11.92, 17.49]	**0.010**
	PINP intact, ng/mL	39.83 [31.49, 47.86]	55.87 [47.40, 68.74]	**0.002**
	TRACP5b, U/L	2.78 [1.84, 3.67]	2.96 [2.42, 3.60]	0.237

Bold numbers indicate statistical significance at *p* < 0.05

**Table 3:** Clinical data, mineral and bone phenotype in ADPKD with CKD G3-G4 vs. CKD G3-G4 of other etiologies

		ADPKD CKD G3-G4 (*n* = 44)	Other etiologies CKD G3-G4 (*n* = 14)	*P* value
	Age, year	47.00 [38.50, 54.00]	40.50 [37.25, 44.25]	0.086
Clinical data	Sex, F/M	14/30 (31.8/68.2)	7/7 (50.0/50.0)	0.361
	BMI, kg/m^2^	26.88 [24.21, 28.98]	23.77 [21.97, 28.73]	0.143
	Creatinine, mg/dL	1.77 [1.48, 2.24]	1.81 [1.63, 2.00]	0.419
	ACR, mg/g	38.30 [11.36, 148.76]	250.36 [42.91, 814.85]	**0.028**
	CRP, mg/dL	1.19 [0.88, 2.74]	1.02 [0.84, 1.57]	0.741
Bone metabolism	Calcium, mg/dL	9.60 [9.40, 9.85]	9.50 [9.22, 9.88]	0.551
	Phosphate, mg/dL	3.10 [2.75, 3.45]	3.15 [2.87, 3.58]	0.413
	Magnesium, mg/dL	1.90 [1.80, 2.10]	1.90 [1.90, 2.00]	0.828
	PTH, pg/mL	56.80 [42.15, 97.55]	55.20 [35.88, 99.05]	0.853
	FGF23 intact, pg/mL	91.97 [61.44, 119.36]	106.78 [79.94, 144.31]	0.367
	FGF23 C-terminal, RU/mL	1.77 [0.82, 2.80]	1.87 [1.58, 3.03]	0.618
	Bioactive sclerostin, pmol/L	85.87 [56.22, 118.78]	57.34 [47.10, 66.34]	**0.042**
	25OHD3, ng/mL	33.01 [22.25, 41.94]	24.75 [19.54, 39.24]	0.392
Bone mass/ microarchitecture	aBMD wb, g/cm^2^	1.15 [1.08, 1.22]	1.15 [1.03, 1.23]	0.786
	TBS	1.34 [1.24, 1.47]	1.47 [1.38, 1.51]	0.082
Bone strength	BMSi	75.05 [67.65, 81.25]	74.30 [72.73, 80.28]	0.870
Bone turnover	ALP, U/L	65.00 [46.50, 75.50]	64.00 [55.75, 71.75]	0.875
	BALP, µg/L	13.21 [9.91, 16.53]	15.77 [13.97, 19.02]	0.080
	PINP intact, ng/mL	40.63 [31.19, 55.02]	66.11 [59.79, 76.37]	**0.007**
	TRACP5b, U/L	2.68 [2.24, 3.46]	3.99 [2.70, 4.20]	0.050

Bold numbers indicate statistical significance at *p* < 0.05

### Comparative Mineral and Bone Phenotypes across Stages of CKD and ADPKD

Mineral and bone phenotypes were characterized within four predefined domains: bone metabolism, bone mass/microarchitecture, bone turnover and bone strength, as explained in Fig. 1. We adopted the heat maps’ integrative approach, allowing for simultaneous visualization of biomechanical and skeletal alterations across disease stages. Analyzes were performed separately according to CKD stage and ADPKD stage (MIC). To facilitate transparency and allow clinical interpretation, absolute values of all measured parameters are presented in the Supplementary Material (Tables S1 and S2), while the main text focuses on standardized comparisons and visualization.

**Fig. 2:**
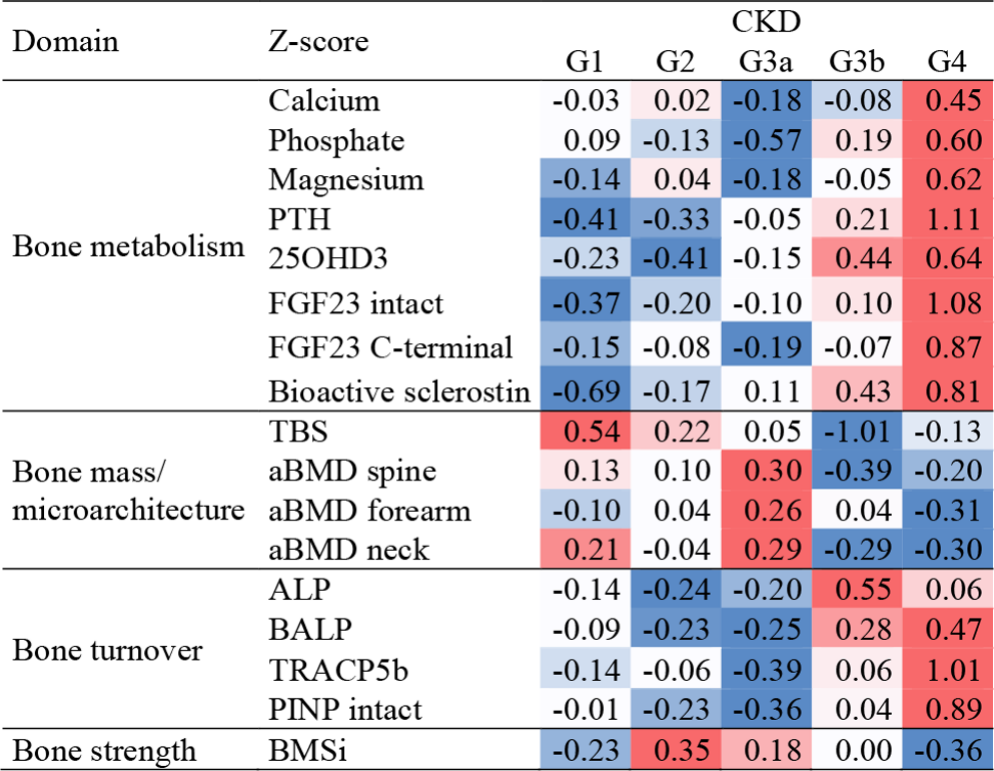
Heatmap of mineral and bone phenotypes across CKD stages in ADPKD (CGA classification). Values represent standardized Z-scores for predefined domains (bone metabolism, bone mass and microarchitecture, bone turnover, bone strength). Red colors indicate higher values and blue colors indicate lower values. Note: Heat maps show standardised trends (z-scores) and may visually exaggerate small numerical differences. Absolute values and statistical results are provided in the Supplementary Tables

Across CKD stages, within the ADPKD cohort, patients displayed progressive shifts in mineral and bone parameters (Fig. 2). In early stages (G1–G2), circulating minerals (phosphate, magnesium) and mediators of bone metabolism (PTH, FGF23, bioactive sclerostin) were generally reduced, bone mass and microarchitecture were preserved, and bone turnover markers (PINP, BALP, TRAcP5b) were consistently suppressed. With disease progression, a marked transition occurred. At stage G3b–G4, phosphate, PTH, FGF23 and sclerostin increased substantially, accompanied by a rise in bone turnover markers (ALP, BALP, TRACP5b, PINP). In parallel, bone mass and microarchitecture declined, with the most pronounced reduction observed in spine aBMD and TBS. Overall, the pattern suggests that the intrinsic skeletal phenotype of ADPKD may become progressively masked by the dominant features of CKD-MBD as kidney dysfunction advances.

**Fig. 3:**
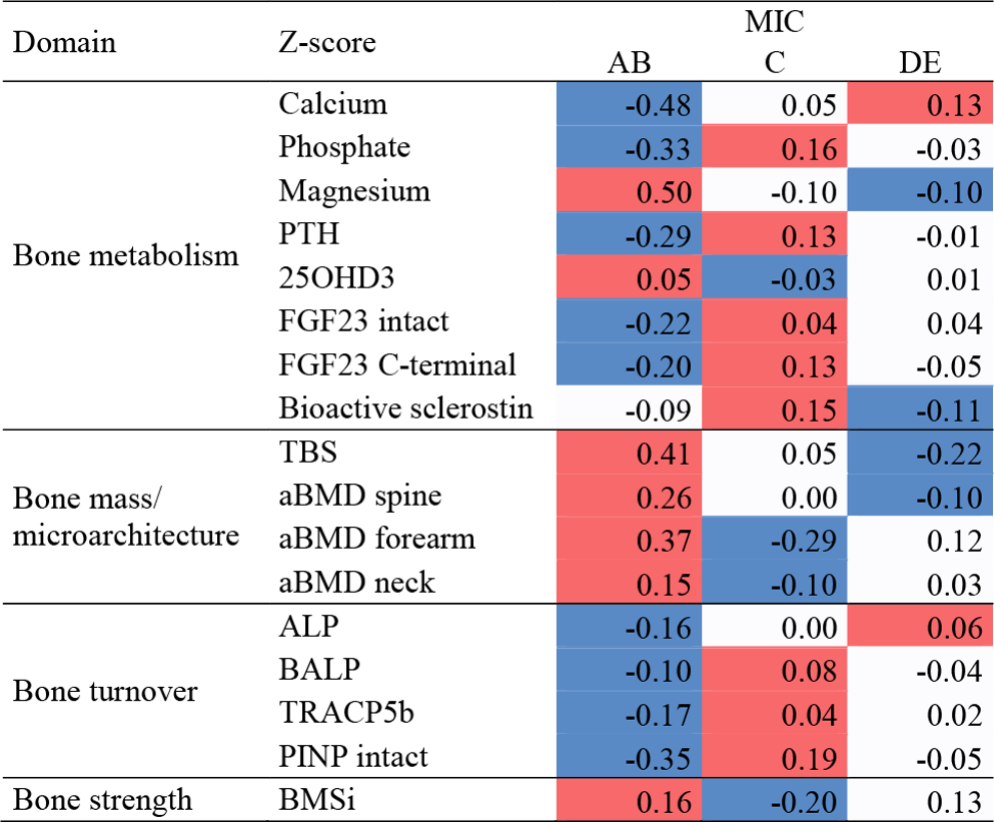
Heatmap of mineral and bone phenotypes across Mayo imaging classification (MIC) stages in ADPKD. Original MIC categories (1 A–1E) were grouped into three strata (AB = low risk, C = intermediate risk, DE = high risk of disease progression). Values represent standardized Z-scores for predefined domains (bone metabolism, bone mass and microarchitecture, bone turnover, bone strength). Red colors indicate higher values, while blue colors indicate lower values. Note: Heat maps show standardised trends (z-scores) and may visually exaggerate small numerical differences. Absolute values and statistical results are provided in the Supplementary Tables

For analysis according to MIC, patients were grouped into three clinically coherent risk strata (AB = low, C = intermediate, DE = high progression risk), and heatmap analysis demonstrated progressive alterations across categories (Fig. 3). In AB, mineral and bone parameters were largely preserved. In C, early abnormalities became evident, including reductions in aBMD and TBS, higher sclerostin, and increased bone turnover markers. In DE, disturbances intensified with pronounced loss of bone mass and further increases in turnover. BMS showed a variable pattern, with the highest values in AB, a marked reduction in C, and partial recovery in DE.

### Independent Determinants of Bone Phenotype in ADPKD

To assess how kidney disease progression influences bone phenotype in ADPKD, we evaluated the relative contribution of MIC and CKD stages, along with biochemical and demographic predictors, to variation in bone-related parameters. A rank-based linear regression model was used to determine which variables most strongly corresponded with differences in bone metabolism, turnover, mass, and strength across disease stages. Bone metabolism markers contributed variably to the overall bone phenotype in ADPKD. When disease severity was expressed according to CKD stages, intact FGF23 showed the strongest association (5.7% of the total feature importance), whereas ALP and bioactive sclerostin contributed only marginally (< 1%). In contrast, when disease severity was assessed using MIC classification, a more pronounced bone-related pattern emerged: intact FGF23 (12.9%) and C-terminal FGF23 (11.6%) were among the top determinants, with bioactive sclerostin contributing 6.0%.

## Discussion

This is the first study that comprehensively investigates the mineral and bone phenotype of ADPKD across the spectrum of CKD and MIC stages. We identified a bone phenotype with reduced bone turnover markers and lower circulating phosphate and magnesium in ADPKD patients with preserved kidney functions, compared to healthy individuals. Reduced bone turnover markers together with increased slerostin characterized also the bone phenotype in ADPKD patients with CKD G3-4, compared to non-ADPKD patients with CKD G3-4.

Polycystin expression in osteocytes [16], osteoclasts [17], and the extracellular matrix (ECM) [18] offers a biological basis for impaired bone quality in ADPKD. Preclinical studies further support this by indicating disrupted bone turnover and mineralization in models of ADPKD [14].

Ourfindings of abnormalities in phosphate and magnesium already in early ADPKD compared with healthy controls are consistent with prior studies reporting early mineral abnormalities in this population. Pavik et al. described hypophosphatemia in 38% of adults with ADPKD, associated with markedly elevated FGF23 and reduced tubular maximum of phosphate reabsorption per GFR, despite normal PTH and 25(OH)D levels [15]. Similarly, De Rechter et al. demonstrated lower serum phosphate and reduced phosphate reabsorption in children with ADPKD compared with healthy peers, accompanied by differences in FGF23, Klotho, and bone biomarkers [16]. The nominally higher FGF23 levels in ADPKD patients with preserved kidney function compared to healthy individuals in the current study did not reach significance, possibly due to large variabilities in both groups. Still, together with the earlier studies, our data support the concept that phosphate dysregulation is an early and disease-specific component of the ADPKD phenotype, potentially driven by altered FGF23 signalling. Experimental studies further suggest that polycystin deficiency in osteocytes may influence the regulation of FGF23 and PTH, providing a biological rationale for this hypothesis [17]. When assessed across disease stages, however, the mineral phenotype appeared to follow the progression of CKD rather than the MIC stratification, suggesting that systemic mineral alterations, albeit present early, are more closely linked to further kidney function decline. This finding contradicts the hypothesis that kidney cyst burden contributes to extraosseous FGF23 synthesis, although it does not exclude a potential role of hepatic cysts in this process [4].

Bioactive sclerostin levels in our group of ADPKD patients were elevated, in line with previous observations [2, 3]. Interestingly, this increase was not uniform across stages but became more pronounced with advancing disease, suggesting that bioactive sclerostin upregulation may be a later rather than an early feature of the ADPKD bone phenotype. This pattern resembles findings in other CKD etiologies, where bioactive sclerostin rises progressively with declining kidney function [18], but is consistently disproportionately higher in ADPKD [2, 3]. We hypothesize that in early stages of ADPKD, when renal function remains preserved, kidneys appear capable of effectively clearing excess bioactive sclerostin. However, as the disease progresses and kidney function deteriorates, this clearance mechanism becomes impaired, revealing bioactive sclerostin excess.

Bone morphology and BMD in ADPKD have been reported as relatively preserved compared with non-ADPKD CKD populations when matched for eGFR, with some studies even suggesting slightly higher values at cortical sites [19]. Recent data extend these observations: after kidney transplantation, ADPKD patients demonstrated maintained or improved bone health indices [19]. Moreover, pharmacological treatment with tolvaptan has been associated with alterations in mineral metabolism and increased BMD [20]. However, none of the individuals in our cohort had received tolvaptan. BMD and TBS were relatively preserved in early ADPKD but declined in more advanced CKD and MIC stages, with spine and neck sites most affected. These observations nicely align with prior reports. Taken together, these findings suggest that, while bone turnover is consistently reduced in ADPKD, bone density and morphology may be relatively preserved, potentially reflecting unique disease-related factors that may account for the discrepancy observed between bone turnover and bone mass domains.

BMS assessed by impact microindentation was included in this study as a novel, minimally invasive approach to capture bone material quality beyond density. This is of particular interest in ADPKD, where bone quality may be affected by polycystin expression in ECM and especially in osteoclasts. In our cohort, however, BMS showed only limited differences across stages and groups. Part of this may be attributed to methodological and sample-size limitations discussed below. While the biological rationale remains compelling, the present findings suggest that the role of BMS in ADPKD is less clear than initially anticipated and requires confirmation in larger, dedicated studies.

Compared with healthy controls, ADPKD patients showed evidence of suppressed bone turnover, with significantly lower levels of PINP and BALP. This low-turnover phenotype was then consistently reproduced in disease staging analyses. When we compared both classification systems, the GFR-based CKD staging showed clearer and more consistent changes in bone parameters than the MIC system. Heatmap visualization across both CKD (CGA) and Mayo (MIC) classifications demonstrated a uniform downward shift in markers of bone formation (BALP, PINP) and resorption (TRAcP5b), in contrast to the more variable patterns observed in bone mass and metabolism. These findings strengthen the concept of a low-turnover skeletal phenotype in ADPKD, consistent with earlier reports [2, 21]. Unlike in other CKD etiologies, where advancing renal impairment is typically accompanied by secondary hyperparathyroidism and increased remodeling, ADPKD patients appear to exhibit a relative suppression of bone turnover throughout the disease course. This may reflect mechanisms intrinsic to ADPKD, including altered osteocytic signaling, increased bioactive sclerostin, and experimental evidence of partial resistance to PTH.

Clinically, the observation of consistent low turnover across two independent staging systems highlights that the bone phenotype alterations in ADPKD do not “precede” kidney disease progression, but rather follow CKD severity. This distinction is important, as it suggests that skeletal abnormalities in ADPKD may not be directly linked to cyst burden, but are instead a manifestation of systemic CKD-MBD. Nevertheless, the relative uniformity of suppressed turnover across stages suggests a disease-specific imprint that merits further exploration.

By applying linear regression, we identified parameters with the most pronounced impact on stage-specific differences in the whole ADPKD cohort. The use of a robust regression approach minimized the influence of outliers and ensured consistent detection of these differences. Interestingly, bone metabolism markers (FGF23, bioactive sclerostin) outperformed all other variables, including demographic and clinical parameters, but only when stratified by MIC classification. This pattern was not observed under CKD staging.

This study has several limitations. First, it was conducted in a relatively small cohort from a single center, which may restrict the generalizability of the findings. Second, the diagnosis of ADPKD was established based on clinical and radiological criteria, without systematic genetic confirmation. Third, the cross-sectional design provides only a snapshot of the mineral and bone phenotype and does not allow conclusions regarding causality or longitudinal changes. Fourth, as CKD G1–2 patients were not available as a functional control group, healthy individuals were used for comparison. Unmeasured factors such as other medications, dietary intake, or metabolic modifiers may have introduced residual confounding. Finally, the absence of bone biopsy and advanced imaging techniques such as HR-pQCT limits the resolution of microarchitectural assessment, and the analyses relied on surrogate measures of bone quality.

Measuring bone metabolism and quality, including its mechanical properties, is challenging without a bone biopsy [22]. The limited access to the latter is reflected in the literature, which reports just a few cases of histomorphometry in individuals with ADPKD [2, 21]. This study provides the first comprehensive non-invasive assessment of the mineral and bone phenotype in ADPKD across both CKD and MIC stages. Our results demonstrate a consistent low bone turnover profile, already evident compared with healthy controls and persisting across disease progression. While bone mass appeared relatively preserved, microarchitectural alterations and suppressed remodeling indicate a distinct skeletal phenotype that differs from other CKD etiologies. Importantly, the bone phenotype in ADPKD seems to follow CKD severity rather than cyst burden, underscoring its integration within the broader CKD-MBD spectrum. These findings highlight the need for further mechanistic studies and clinical strategies tailored to the unique bone fragility risk in ADPKD.

## Supplementary Information

Below is the link to the electronic supplementary material.

Supplementary Material 1

## Data Availability

The data supporting the findings of this study are available from the first author upon reasonable request. No publicly unavailable datasets were generated or analyzed during the current study.
