# Supplementary material for: Bone Phenotype in Autosomal Dominant Polycystic Kidney Disease
Source: Calcif Tissue Int. 2026 Jan 23;117(1):13. doi: 10.1007/s00223-025-01471-w (PMC12827306; doi:10.1007/s00223-025-01471-w)
Supplement: Supplementary file 1 — Supplementary Material 1 [file 223_2025_1471_MOESM1_ESM.docx]

**Supplement**

**Table 1S.** Mineral and bone phenotype in ADPKD across stages of CKD.

|  | **ADPKD**  **CKD G1-G2**  **(n = 37)** | **ADPKD**  **CKD G3a**  **(n = 16)** | **ADPKD**  **CKD G3b- G4**  **(n = 28)** | **P value** |
| --- | --- | --- | --- | --- |
| Sex, F/M | 21/16 (56.8/43.2) | 4/12 (25.0/75.0) | 10/18 (35.7/64.3) | 0.062 |
| Calcium, mg/dL | 9.50 [9.30, 9.90] | 9.50 [9.38, 9.72] | 9.60 [9.45, 9.90] | 0.722 |
| Phosphate, mg/dL | 3.25 [2.80, 3.40] | 2.85 [2.60, 3.20] | 3.20 [2.90, 3.70] | **0.016** |
| Magnesium, mg/dL | 1.90 [1.80, 2.00] | 1.90 [1.80, 2.00] | 2.00 [1.80, 2.10] | 0.511 |
| PTH, pg/mL | 34.40 [21.00, 45.10] | 53.85 [33.12, 78.50] | 65.50 [45.90, 117.50] | **<0.001** |
| FGF23 intact, pg/mL | 52.05 [34.92, 64.95] | 67.91 [49.01, 93.19] | 104.05 [76.65, 135.89] | **<0.001** |
| FGF23 C-terminal, RU/mL | 1.04 [0.43, 2.04] | 0.83 [0.58, 1.35] | 2.26 [1.39, 3.64] | **0.001** |
| Bioactive sclerostin, pmol/L | 45.27 [30.74, 62.50] | 73.42 [58.25, 106.01] | 96.69 [56.22, 133.21] | **<0.001** |
| 25OHD3, ng/mL | 25.87 [20.12, 32.49] | 21.94 [15.42, 37.11] | 37.05 [30.91, 43.28] | **0.002** |
| aBMD wb, g/cm^2^ | 1.13 [1.08, 1.18] | 1.22 [1.14, 1.32] | 1.13 [1.07, 1.19] | 0.186 |
| TBS | 1.45 [1.38, 1.51] | 1.44 [1.30, 1.49] | 1.31 [1.21, 1.40] | **0.002** |
| BMSi | 76.10 [64.60, 81.00] | 76.95 [72.57, 81.53] | 73.30 [66.45, 80.08] | 0.754 |
| ALP, U/L | 53.00 [43.00, 63.00] | 59.50 [40.00, 66.00] | 71.00 [53.50, 79.50] | **0.043** |
| BALP, µg/L | 10.87 [9.75, 14.99] | 10.09 [8.64, 15.43] | 13.48 [11.11, 16.72] | 0.054 |
| PINP intact, ng/mL | 39.83 [31.49, 47.86] | 36.23 [28.39, 41.31] | 44.96 [36.06, 65.43] | **0.044** |
| TRACP5b, U/L | 2.78 [1.84, 3.67] | 2.25 [1.96, 3.05] | 2.91 [2.40, 4.15] | 0.066 |

**Table 2S.** Mineral and bone phenotype in ADPKD across stages of MIC.

|  | **ADPKD AB**  **(n = 13)** | **ADPKD C**  **(n = 33)** | **ADPKD DE**  **(n = 35)** | **P value** |
| --- | --- | --- | --- | --- |
| Sex, F/M | 5/8 (38.5/61.5) | 19/14 (57.6/42.4) | 11/24 (31.4/68.6) | 0.087 |
| Calcium, mg/dL | 9.40 [9.20, 9.50] | 9.50 [9.30, 9.90] | 9.70 [9.50, 9.90] | 0.063 |
| Phosphate, mg/dL | 3.00 [2.90, 3.30] | 3.20 [2.90, 3.50] | 3.20 [2.70, 3.50] | 0.350 |
| Magnesium, mg/dL | 2.00 [1.90, 2.10] | 1.90 [1.80, 2.00] | 1.90 [1.80, 2.00] | 0.195 |
| PTH, pg/mL | 38.50 [19.90, 43.50] | 52.00 [33.80, 72.40] | 47.05 [28.92, 71.15] | 0.200 |
| FGF23 intact, pg/mL | 55.93 [34.92, 73.93] | 55.66 [43.15, 82.17] | 84.52 [63.03, 117.60] | **0.007** |
| FGF23 C-terminal, RU/mL | 0.64 [0.45, 1.25] | 1.06 [0.81, 2.57] | 1.93 [1.10, 3.53] | **0.017** |
| Bioactive sclerostin, pmol/L | 57.12 [50.49, 70.69] | 63.00 [45.91, 105.46] | 57.14 [33.71, 89.15] | 0.417 |
| 25OHD3, ng/mL | 27.95 [22.16, 35.40] | 30.76 [21.30, 39.01] | 26.87 [20.63, 37.82] | 0.974 |
| aBMD wb, g/cm^2^ | 1.15 [1.13, 1.31] | 1.09 [1.00, 1.15] | 1.17 [1.11, 1.22] | **0.033** |
| TBS | 1.45 [1.39, 1.52] | 1.43 [1.33, 1.50] | 1.35 [1.28, 1.45] | 0.092 |
| BMSi | 76.60 [69.90, 80.50] | 74.00 [62.10, 81.40] | 75.70 [69.40, 80.35] | 0.530 |
| ALP, U/L | 54.00 [46.00, 65.00] | 58.00 [43.00, 69.00] | 61.00 [49.50, 75.75] | 0.703 |
| BALP, µg/L | 11.71 [9.76, 15.20] | 10.98 [9.71, 14.99] | 13.30 [10.06, 16.19] | 0.690 |
| PINP intact, ng/mL | 38.38 [29.66, 39.28] | 39.15 [31.47, 48.06] | 42.85 [32.72, 55.11] | 0.131 |
| TRACP5b, U/L | 2.68 [1.96, 3.30] | 2.32 [2.04, 3.27] | 2.78 [2.20, 3.67] | 0.873 |

**Figure 1S.** ADPKD with preserved kidney function (CKD G1-G2) vs. healthy.

**
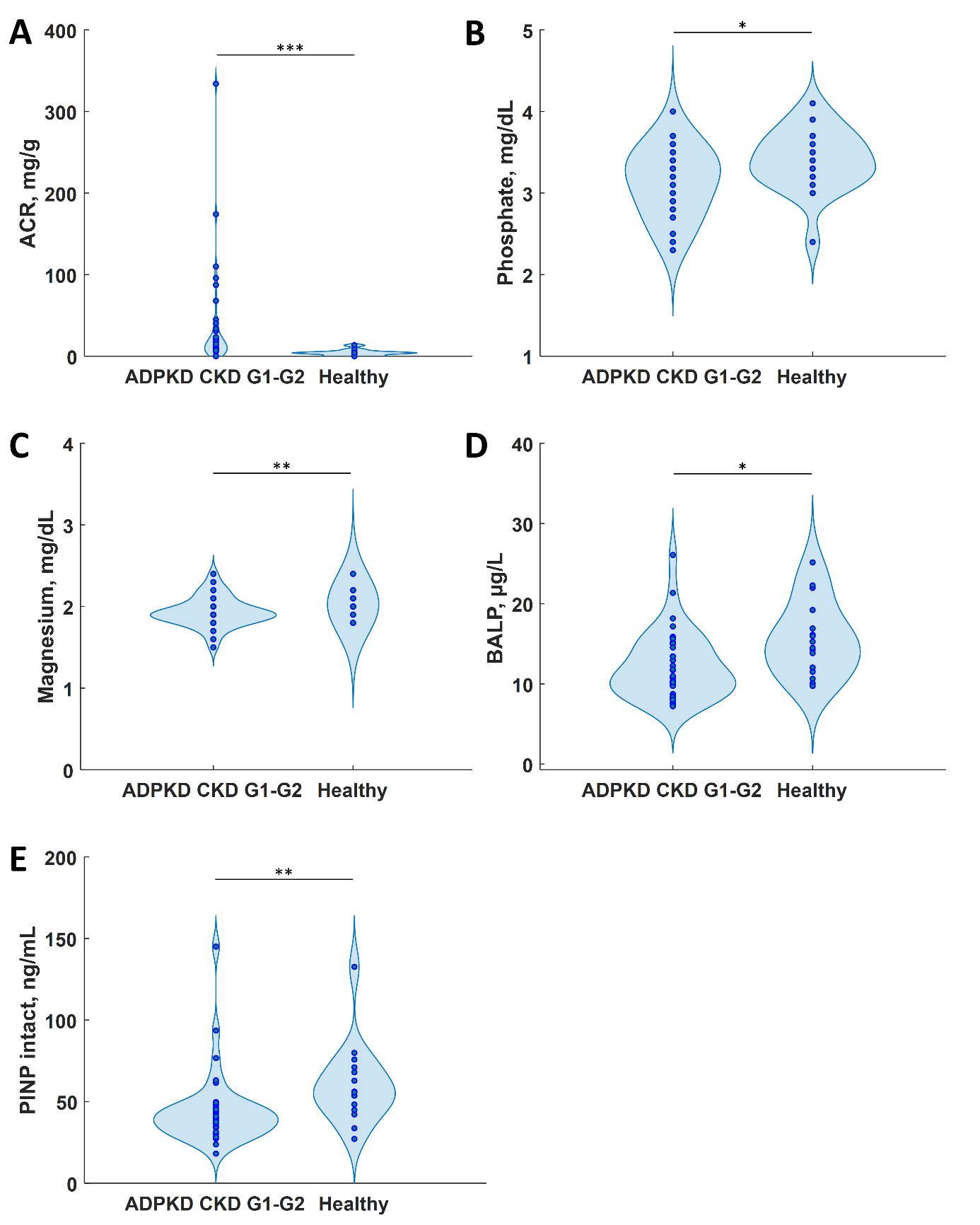
**

**Figure 2S.** ADPKD with CKD G3-G4 vs. CKD G3-G4 of other etiologies

**
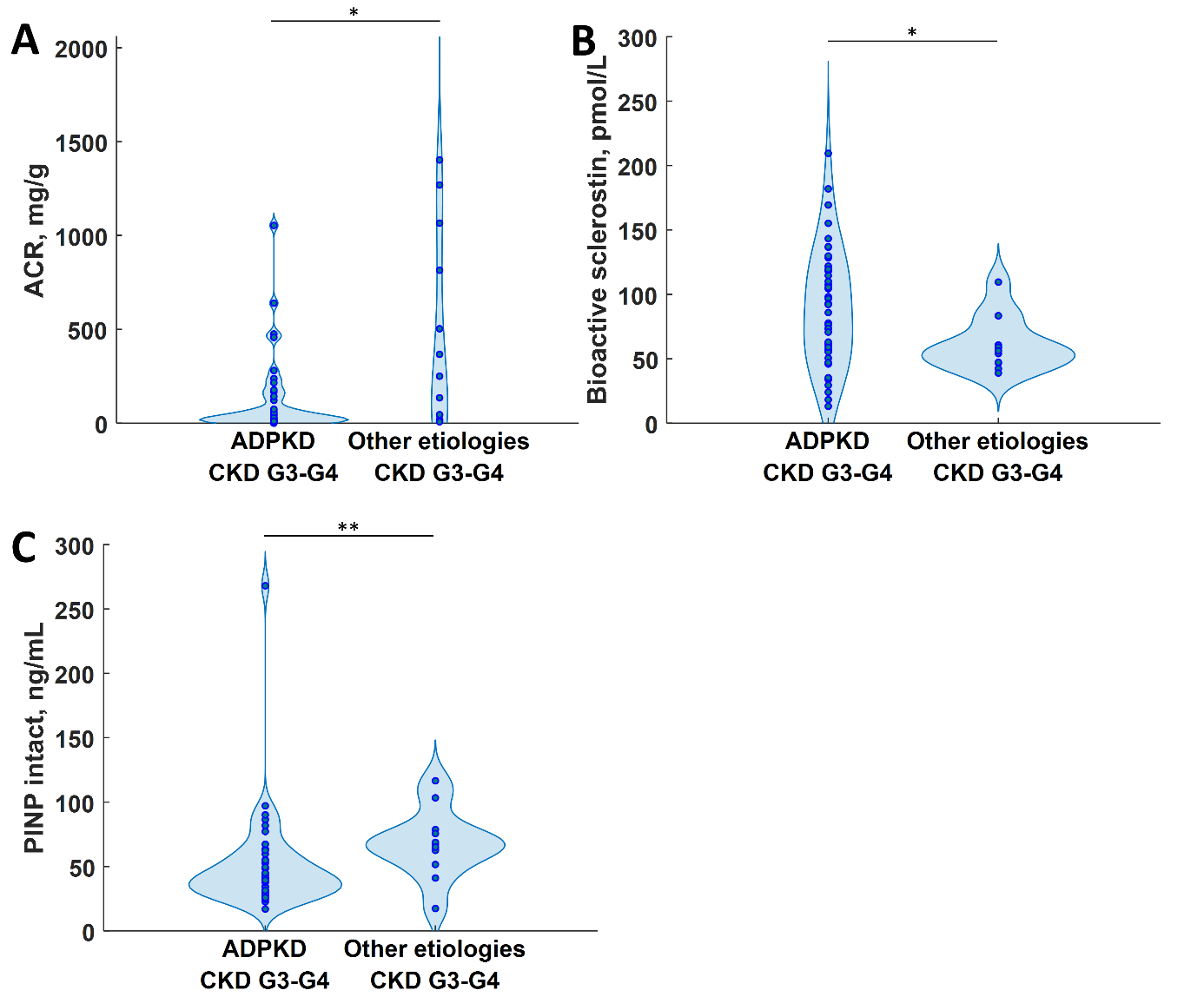
**
